# Ultrashort Sintering and Near Net Shaping of Zr-Based AMZ4 Bulk Metallic Glass

**DOI:** 10.3390/ma14195862

**Published:** 2021-10-07

**Authors:** Łukasz Żrodowski, Rafał Wróblewski, Tomasz Choma, Tomasz Rygier, Marcin Rosiński, Bartosz Morończyk, Maweja Kasonde, Marcin Leonowicz, Jakub Jaroszewicz, Mateusz Ostrysz, Wojciech Łacisz, Piotr Błyskun, Karolina Pomian

**Affiliations:** 1 Faculty of Materials Science and Engineering, Warsaw University of Technology, Woloska 141 St., 02-507 Warsaw, Poland; tomasz.rygier.dokt@pw.edu.pl (T.R.); bartosz.moronczyk.dokt@pw.edu.pl (B.M.); marcin.leonowicz@pw.edu.pl (M.L.); jakub.jaroszewicz@pw.edu.pl (J.J.); Piotr.Blyskun@pw.edu.pl (P.B.); kp.pomian@wp.pl (K.P.); 2 R&D Department, AMAZEMET Sp. z o.o., Al. Jana Pawła II 27, 00-867 Warsaw, Poland; mateusz.ostrysz@amazemet.com (M.O.); wojciech.lacisz@amazemet.com (W.Ł.); 3 R&D Department, GeniCore Sp. z o.o., 133 Wolczynska Street, 01-919 Warsaw, Poland; Marcin.Rosinski@genicore.pl (M.R.); Maweja.Kasonde@genicore.pl (M.K.)

**Keywords:** spark plasma sintering, bulk metallic glasses, rapid sintering, SPS

## Abstract

The GeniCore Upgraded Field Assisted Sintering Technology U-FAST was applied to the sintering of a commercial Zr-based bulk metallic glass powder AMZ4. The XRD, SEM and DSC analysis of the sintered compacts showed the benefit of the U-FAST method as an enabler for the production of fully amorphous samples with 100% relative density when sintering at 420 °C/480 s (693 K/480 s) and 440 °C/ 60 s (713 K/480 s). The hardness values for fully amorphous samples, over HV1 519, surpass cast materials and 1625 MPa compressive strengths are comparable to commercial cast products. The advantage of the U-FAST technology in this work is attributed to the high heating and cooling rates inherent to ultra-short pulses, which allow to maintain metastable structures and achieve better temperature control during the process. Increasing sintering temperature and time led to the crystallization of the materials. The geometry and material of the dies and punch determine the thermal inertia and pressure distribution inside the compacts, thus affecting the properties of the near net shape NNS compacts made using the U-FAST device.

## 1. Introduction

Spark Plasma Sintering (SPS)—which is also referred to as Field Assisted Sintering Technology (FAST)—is a sintering technology that is becoming increasingly important in the processing of various materials, such as bulk metallic glasses, composite materials, nanostructured materials and gradient materials. The basic theory of SPS/FAST heating infers that the pulsed current flows through the mould and causes localized heating of the surface of the initial powder particles. The current flow generates an electric field with a plasma effect throughout the sintered volume [1,2]. The FAST/SPS technology offers some benefits over the standard vacuum sintering followed by hot isostatic pressing (HIP) by the single configuration required for densification, and the shortened sintering cycles. Nevertheless, the initial powder quality and homogeneous packing are crucial for the density of the manufactured sample [3].

Various bulk metallic glasses (BMGs) have already been successfully developed in various alloy systems, such as Mg-Cu-Y, La-Al-Ni, Zr-Al-Ni-Cu, Zr-Al-Ni-Cu, Zr-Ti-Cu-Ni-Be, Ti-Ni-Cu-Sn, Cu-Zr-Ti-Ni, Nd-Fe-Co-Al, Fe-Co-Ni-P, refs. [4,5,6,7,8,9,10], etc. However, BMGs are difficult to produce due to the high cooling rate requirement for amorphous solidification, which still limits their producible size and, therefore, their broader application. Recent works have brought significant progress in the field of sintering activated by electric fields [11]. This activation method allows the sintering process to be carried out in a very short time: from a few seconds to several minutes. This, in turn, limits the extent of diffusion processes, thus maintains the non-equilibrium structure of the material and prevents grain growth processes in the consolidated product. The compaction under an external load applied following an appropriate load–time profile can reduce the porosity of the processed samples [12]. Bulk metallic glasses, due to their high mechanical strength, good fracture toughness, high hardness and superior corrosion resistance [13] are especially promising for this technique. Numerous attempts have been made to overcome the critical casting diameter by sintering powders in the solid or supercooled liquid state; however, the compromise between porosity and maintaining the amorphous structure is still challenging [11,12,13,14,15]. Process control is achieved by changing the sintering temperature or time while the current pulsation frequency is kept constant.

Louzguine-Luzgin [16] has suggested that the crystal nucleation in MG is heterogeneous and that the nucleation sites probably pre-exist in amorphous alloys. Paul and Harimkar [17] have shown that a faster heating rate led to lower temperature densification of Fe_48_Mo_14_Cr_15_Y_2_C_15_B_6_ metallic glass. It is shown that a faster heating rate enables lower temperature densification. The authors reported a second densification event observed at a temperature largely above the crystallization temperature_._

Contrary to Fe-based MG, Zr-based amorphous alloys are commonly produced by mould casting. Some of them are even commercialized (Vitreloy alloys, Materion Performance Alloys and Composites, Ohio, Mayfield Heights). They are thermally stable and often present a wide supercooled liquid region. The literature on Zr-based amorphous alloys is quite limited, though this group of alloys represents a good base for studying the process or structural evolution of the alloys during SPS. Works by Xie et al. [18,19,20] focused on manufacturing porous materials at low sintering temperatures (T_s_ < 0.9 T_g_) without devitrification of the starting material. These authors have reported a decrease in apparent elastic moduli of Zr-based amorphous alloys with increasing porosity content.

The present study investigated the influence of ultrashort pulses compared to standard SPS systems on the compaction of Zr-based bulk metallic glass.

## 2. Materials and Methods

**Figure 1:**
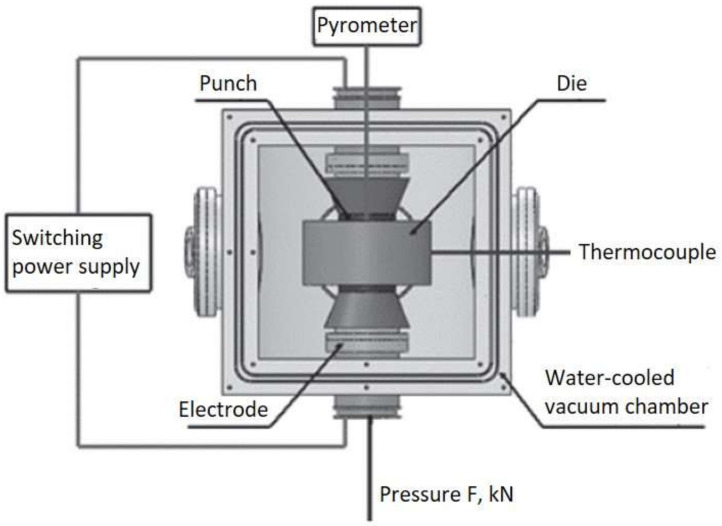
U-FAST diagram.

Gas atomized amorphous AMZ4 powder, provided by Heraeus GmbH, Hanau, Germany, with a particle size distribution of 15–45 µm was used as a feedstock material. Sintering experiments were performed using a novel U-FAST GeniCore (Warsaw, Poland) Upgraded Field Assisted Sintering Technology (U-FAST) SPS schematized in Figure 1. The U-FAST technology allows for full densification in a much shorter period of time and lower temperature when compared to the other sintering methods [21,22]. On the contrary, to standard SPS, the U-FAST device has a pulse duration shorter than 1 ms and operates in a high vacuum. According to the manufacturers’ data sheets, the glass transition temperature T_g_ is 400 °C (673 K) and the crystallization temperature T_x_ is 475 °C (748 K) [23]. This characterization was confirmed by differential scanning calorimetry. In this temperature range, pronounced plastic behavior of the alloy and accelerated densification are to be expected. Powder mixtures were placed into cylindrical graphite die with an inner diameter of 20 mm. Thin graphite sheets and discs were used as liners between the powders and the die walls and punch surfaces. Sintering was conducted at a high vacuum (10^−5^ mbar). The pulse sequence was set to 1:1 (t_ON_:t_OFF_) with 800 µs duration. The temperature was monitored by axial and side optical pyrometers, and for the sintering process of the near net-shaped specimen, an additional thermocouple in a form was used to achieve more precise temperature control. The sintering profiles comprised the following steps: (i) Heating up to 100 °C (373 K) in 2 min. (ii) Dwell at 100 °C for 2 min (373 K). (iii) Heating up to the sintering temperatures of 380; 400; 420; 440; 460 °C (653; 673; 693; 713; 733 K) with the rate of 100 °C/min (373 K/min). (iv) Dwell for 1 min or 8 min at the sintering temperature. (v) Cooling down to room temperature in 8 min. All samples were sintered at a pressure of 60 MPa. Sintered samples were cut on w-EDM machine and prepared for metallographic observation with standard methods. The porosity of the sintered discs was determined by the Archimedes method and the porosity of the gear wheel was measured by computer tomography on Xradia XCT-400 (Hamamatsu, Japan). The changes in the sample with the highest density were analyzed with reference to the raw powder material by DSC and XRD methods. DSC measurements were performed with a heating rate of 40 K/s on Perkin Elmer DSC 8000 (Waltham, MA, USA), while XRD was performed in a Rigaku Miniflex II within the two theta range of 20–70°. That sample was also cut to a 3 mm diameter and 4.5 mm height cylinder and compressed on Instron 20 universal testing machine with a speed of 5 × 10^−4^ 1/s. Hardness was tested using the Vickers method under 1 kgf load.

In addition, a set of punches and a die in the design of a gear wheel with an external diameter of 25 mm was made to assess the net formability of the alloy. The die was manufactured via wire electric discharge machining technique (w-EDM) from Inconel 625 alloy due to its high resistivity and high creep resistance. The punches had an inner part of Inconel 625 and a stud, and an outer punch of Ampcoloy 972.

**Figure 2:**
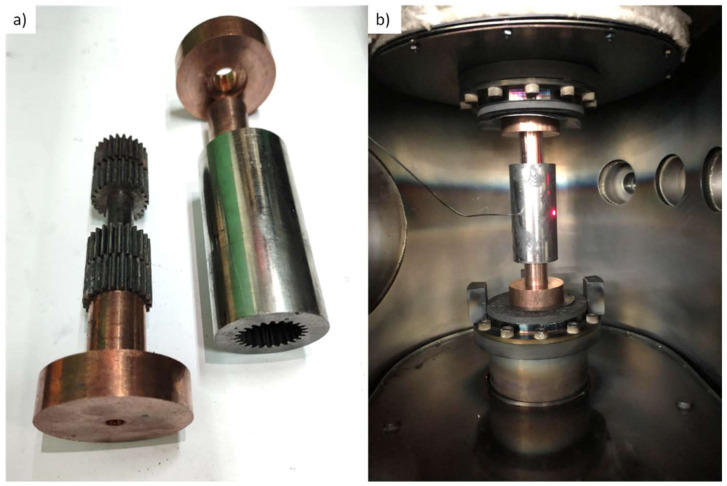
Die assembly for gear wheel sample manufacturing (**a**) an exploded view of the die assembly and (**b**) processing die in U-FAST system.

The inner surfaces were coated with oil-free graphite spray. The difference between thermal and electrical conductivities was designed to heat the die instead of the punches and provide high cooling rates. An exploded view of the die assembly is shown in Figure 2a, while the assembly in U-FAST is shown in Figure 2b.

## 3. Results and Discussion

### 3.1. Manufacturing Process

**Figure 3:**
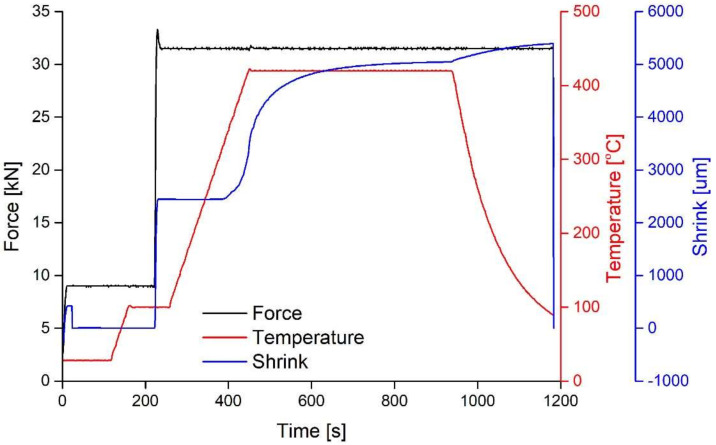
U-FAST process readout.

Figure 3 illustrates a typical shrinkage curve along with the applied load and temperature profiles for a long sintering cycle, 1200 s, of Zr-based metallic glass samples in the U-FAST machine. The two-stage pressure and temperature increases allowed the proper degassing of the samples at a high vacuum. The magnitude of shrinkage red on the recorded curve relates to transformations of the material during sintering. Thus, the shrinkage curve in Figure 3 infers that the Zr-based metal glass powders underwent a transformation at the temperature range 250–400 °C (523–673 K). This characteristic behaviour was observed in all samples sintered in this work. Further heating above this range induced more shrinkage. The inflexion point in the shrinkage curve indicates that the transformation rate during sintering increased with time to a maximum, then it decreased. It may also be relevant to the onset of a different transformation process. The curve also suggests that the sintering process was not completed as the samples still shrunk after 1200 s at the dwell temperature of 400 °C (673 K). 

**Figure 4:**
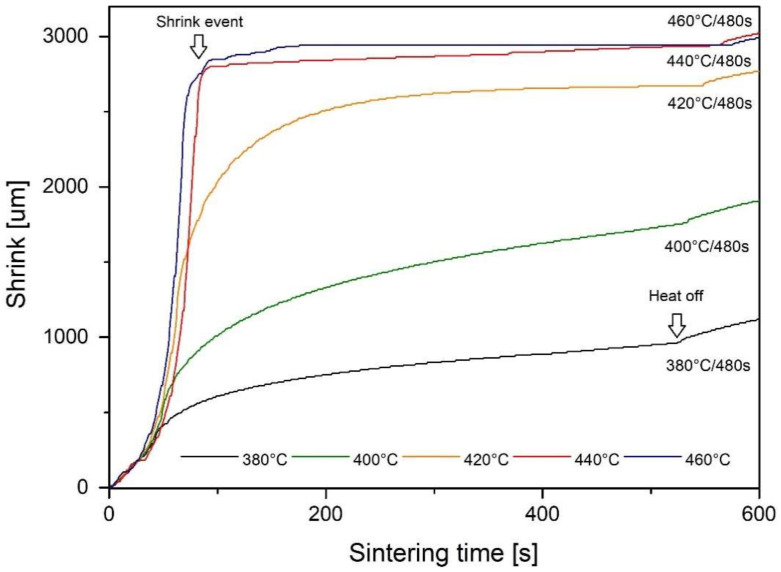
Punch displacement for 480 s sintering time.

**Figure 5:**
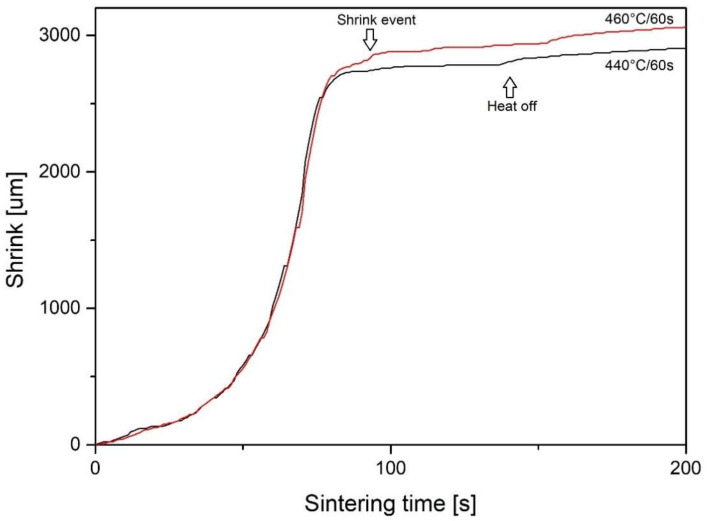
Punch displacement curves for 60 s sintering time.

Figure 4 and Figure 5 represent the shrinkage, load and temperature profiles for shorter sintering cycles of a total of 480 s and 60 s, respectively, and various dwell temperatures. The curves of shrinkage values of Figure 4 reveal incomplete sintering processes in samples sintered with the dwell temperatures 380 °C (653 K) and 400 °C (673 K), whereas shrinkage curves smoothed after dwelling at 420 °C (693 K) for 200 s. The relative positions of the curves in Figure 4 infer that the increase in dwell temperature improved the compaction of the samples and reduced the porosity contents. This view is later discussed using micrography analysis and density measurement. The sample sintered at 440 °C (713 K) shows a very different behavior as sintering tends to be step-wise with minor punch displacement after initial shrink. The sample sintered at 460 °C (733 K) has another step (marked as a “shrink event”) associated with devitrification.

Since a shorter sintering time is desired in commercial applications, samples with a sintering temperature of 440 °C (713 K) and 460 °C (733 K) were processed again with a shorter time of 60 s. A shrink event is still observed at 460 °C (733 K) after 80 s (Figure 5).

**Figure 6:**
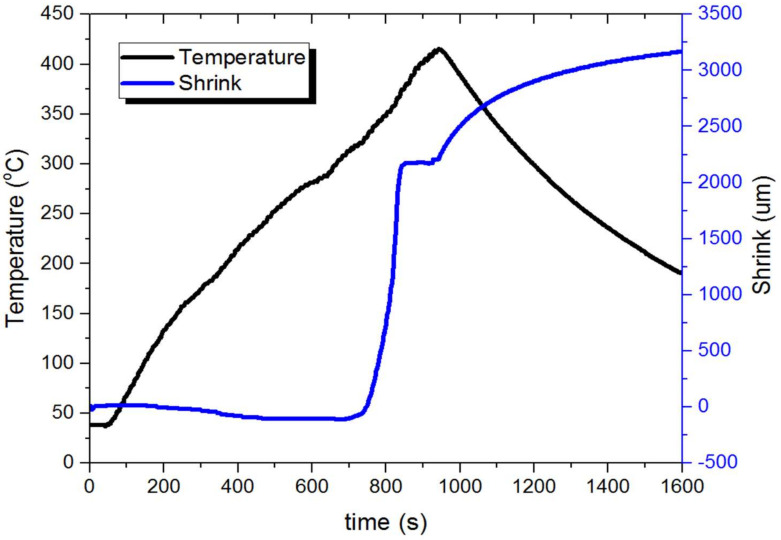
U-FAST process readout for gear wheel sample.

Due to the satisfactory results of the tests on disc-shaped samples, we decided to repeat the best process (Figure 6) from the previous ones on the gear wheel-shaped form. It was intended to present a near net shape sintering demo, which is a challenge in SPS technologies. Subsequent sintering conditions for making a near net shape gear wheel were selected as follows: heating rate of 50 °C/min up to 420 °C (693 K). The process was stopped at 420 °C (693 K) due to the thermal inertia of the system, which meant that the temperature inside was much higher. The readout profiles of the process are shown in Figure 6.

### 3.2. Sample Characterization

**Figure 7:**
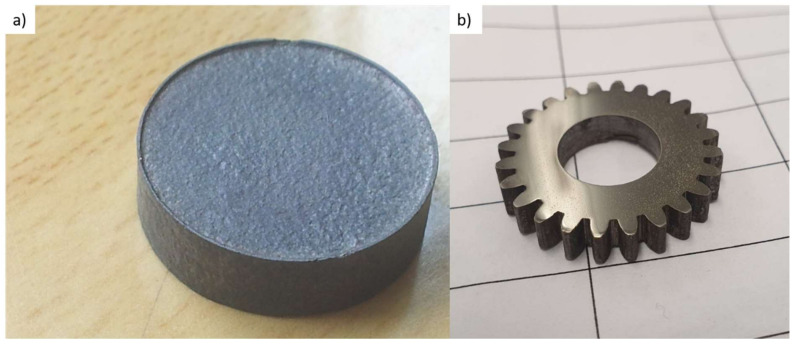
Manufactured samples (**a**) disc and (**b**) gear wheel.

For optimization of the process parameters, the samples were made in the form of simple discs with an outer diameter of 20 mm (Figure 7a). After testing the samples, the best process parameters were chosen and to evaluate net shaping possibility sample in the shape of gear wheel was also manufactured (Figure 7b).

#### 3.2.1. Microstructural Characterization

**Figure 8:**
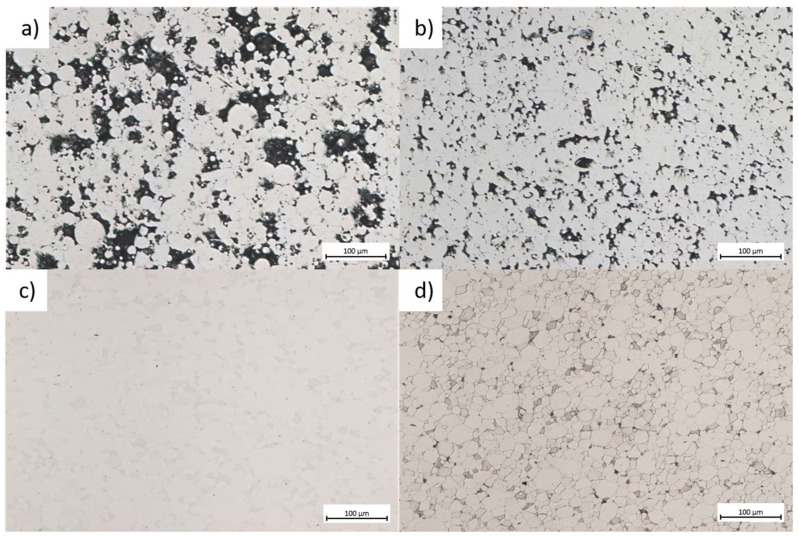
Typical microstructures of sintered materials (**a**) 380 °C/480 s (**b**) 400 °C/480 s (**c**) 420 °C/480 s (**d**) 440 °C/480 s.

The microstructure of the sintered samples is shown in Figure 8. Both 380 °C (653 K) and 400 °C (673 K) samples are highly porous and have only 68.2% and 85.9% density of the cast glassy alloy (Figure 8a,b). The neck formation and internal particle porosity are visible. No particle deformation was observed. Samples sintered at temperatures of 420 °C/480 s (693 K/480 s) and 440 °C/480 s (713 K/480 s) exhibit minimal porosity. Even after etching with a mixture of HF / HNO_3_ no visible discoloration appeared (Figure 8c,d). However, samples processed at higher temperatures showed high crystallization as the etched surfaces tended to blur. Some of the particles crystallized earlier than others, which may be attributed to the chemical inhomogeneity of powder. Moreover, the smallest particles are prone to premature crystallization.

**Figure 9:**
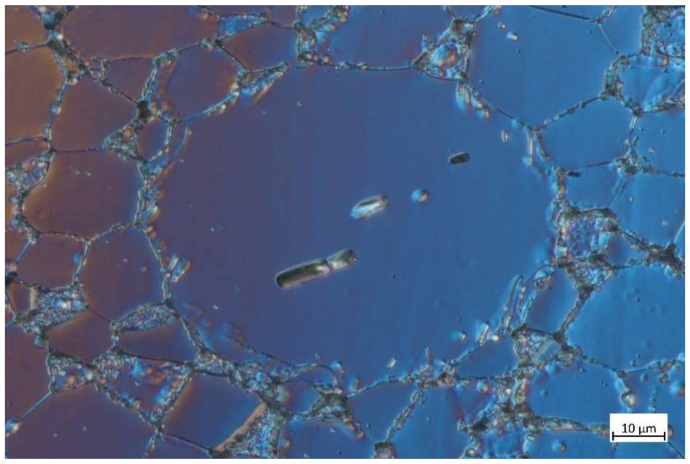
Interparticle porosity of 420 °C/480 s sample.

The interparticle porosity is shown in Figure 9. One can also see that crystallization is strong on the boundary surface of a single particle, which may be related to the mechanism described by Loic et al. [24], where local overheating during necking promotes surface crystallization of the particles.

#### 3.2.2. Computer Tomography

**Figure 10:**
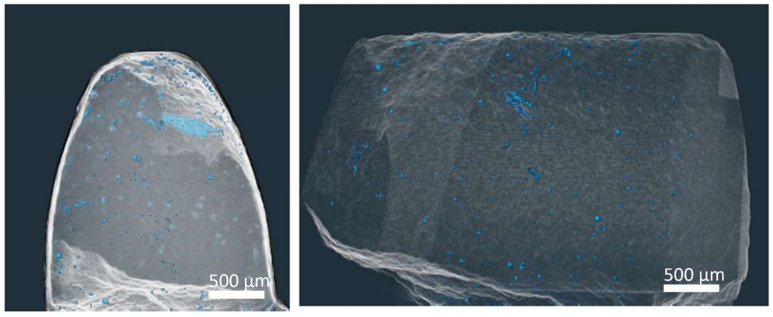
3D models of computer tomography of the gear wheel tooth.

The computer tomography of the gear wheel tooth is shown in Figure 10. Most of the pores are located at the edge of the sintered sample. The total porosity of the sample was estimated with CT software at 0.037% with a pore size distribution of 12–50 µm and a mean size of 17 µm. This may be ascribed to cold sintering at 420 °C (693 K), pressure loss due to friction of the powder particles on the surfaces of the preshaped dies or incomplete filling of the small features with relatively coarse particle size powder. It should also be taken into consideration that the purchased powder contained gas inclusions from atomization process, which are not completely reduced in the sintering process at a given temperature.

#### 3.2.3. XRD Phase Analysis

**Figure 11:**
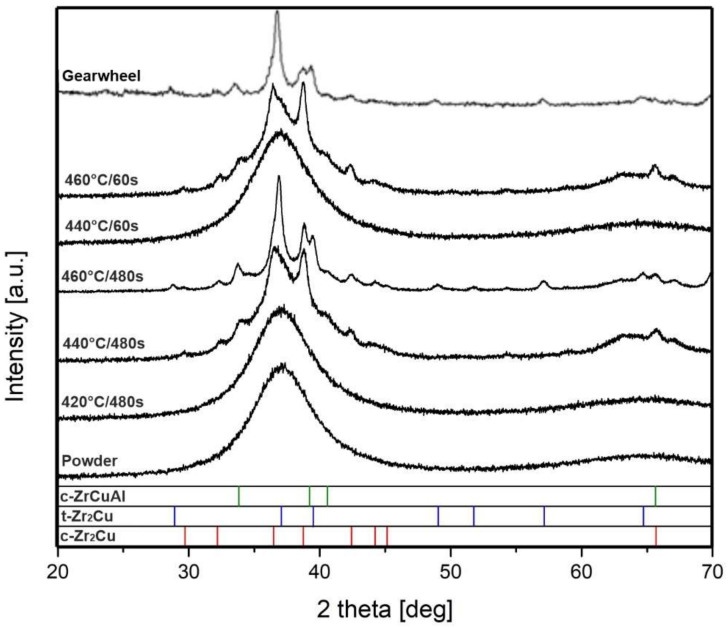
XRD patterns of raw powder, samples sintered at different temperatures and times of 480 s and 60 s and gearwheel sample.

The XRD diffractograms in Figure 11 infer that the material compacted at 420 °C/480 s (693 K/480 s) and at 440 °C/60 s (713 K/60 s) remained amorphous. Increasing the temperature or the time beyond those conditions led to the formation of cubic phase Zr_2_Cu PDF [01-074-7476] and three-component cubic phase ZrCuAl [04-018-3885] or [04-020-8421]. The diffraction peaks of tetragonal phase Zr_2_Cu [04-015-7358] are detected in materials treated at 460 °C (733 K). As shown by Hao et al. [25] electric pulses can induce non-equilibrium phases, other peak values are assigned to such phases which could not be clearly identified. As can be seen in the XRD diagram, the sample in the form of gear was crystalline. It was apparently caused by exceeding the crystallization temperature in the sample, which was overstepped due to the thermal inertia of such a large sintering system. The XRD patterns for the samples sintered at 380 and 400 °C (653 and 673 K) were fully amorphous and very similar to the samples sintered at 420 °C (693 K) and are therefore not shown in the diagram.

#### 3.2.4. DSC Thermal Analyses

**Figure 12:**
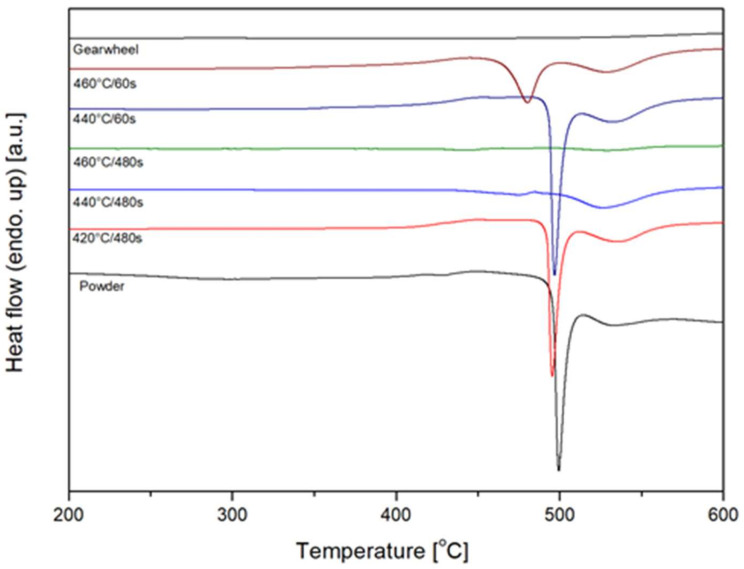
DSC patterns of raw powder and sintered samples.

Differential scanning calorimetry results are shown in Figure 12. Initially, DSC curves of 420 °C (693 K) sample and raw powder do not show any clear difference. In both cases, the calculated enthalpy of crystallization was 57 ± 2 J/g (Table 1) at a crystallization temperature of 496 °C to 500 °C (769 to 773 K). Minor difference in the shape of the peaks was attributed to the different thermal conductivity of the sample (bulk and particulate body). Significant differences can be observed at temperatures above the glass transition point −400 °C (673 K). The bulk sample showed no significant effects, while the raw powder showed a visible relaxation effect, followed by a small endothermic event. The point of inflection (T_c_) was calculated at 309 °C (582 K) which is the same temperature as the offset in the shrink curve (Figure 4).

Let us mention that the sintering temperature of 420 °C (693 K) seemed to be a limit, above which samples crystallized. This 420 °C (693 K) value is apparently low compared to T_x_ determined by the DSC studies. To explain this, three reasons can be invoked:

(1) The processing temperature is in fact higher than that actually measured by the thermocouple, even if it is really close to the sample. However, the electrical current generated during processing, both in graphite mould and powder, may induce a difference between the temperature values inside and outside the mould. In addition, the temperature can be strongly inhomogenous inside the compact as the local electrical resistance depends on the particles’ contact surface area. Therefore, a weak contact results in high local temperature and vice-versa.
(2) Sintering is an isothermal process performed for 5 min in T_s_, while DSC experiments are performed during continuous heating. Since crystallization is a time and temperature-dependent kinetic phenomenon, it can occur at temperatures lower than T_x_ due to the influence of time.
(3) During the whole sintering cycle, a pressure of 60 MPa is used. On one hand, the crystalline phase is slightly denser than its glassy counterpart, thus higher pressure should promote crystallization. On the other hand, the pressure tends to limit the diffusion of atoms, usually necessary for crystallization in BMGs. For this reason, the formation of crystalline particles should be delayed. Therefore, this influence is difficult to unveil, yet it still might have occurred.

#### 3.2.5. Mechanical Properties

Mechanical test results of the studied samples are listed in Table 2. Relative density and other properties were compared with the cast lab-grade materials (Heraeus). From all completely amorphous sintered samples, the sample 440 °C/60 s (713 K/60 s) showed both the highest density and strength. It was on a comparable level with the cast material. The partially crystalline samples showed higher density than the amorphous ones (more than 100%) obviously due to the phase transition. The amorphous phase content was calculated from the proportion of crystallization enthalpy.

**Figure 13:**
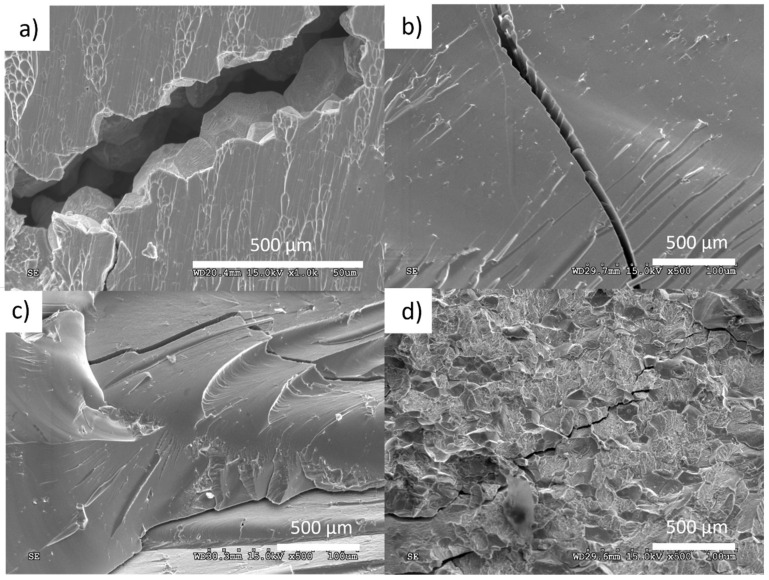
Fractography of sintered samples (**a**) 420 °C/480 s (**b**) 440 °C/480 s (**c**) 440 °C/60 s (**d**) 460 °C/60 s.

Fracture surfaces of all sintered materials are shown in (Figure 13a–d). The materials with 100% amorphous content showed a typical vein pattern on the main fracture surface with zig-zag cracks perpendicular to the main fracture plane. The 420 and 440 °C/480 s, as well as 440 and 460 °C/60 s (733 K/60 s) samples, showed both “intergranular” cracks (Figure 13a) and vein pattern (Figure 13a,c,d). As shown by Nowak et al. [24] the “intergranular” interface can be weakened by oxides present on the initial powder particles’ surface. For the samples sintered at 380 and 400 °C (653 and 673 K), neither the hardness nor the compressive strength could be accurately measured. Due to the porosity of the samples, handling of the sample led to cracking and disintegration, hence further tests were not possible. The higher hardness results (Table 2) of the samples compared to the manufacturer’s cast sample result from the structure of the material. Metallic glasses have an amorphous structure, which is characterized by a denser packing of atoms and the lack of translational symmetry in atomic structures, which results in the absence of expanded defects occurring in crystalline metals. The actual defects in the BMG are much smaller and only become active when greater stress is applied.

## 4. Conclusions

The results of this work highlighted the benefit of the U-FAST technology over the current FAST technology reported in [7] for sintering Zr-based AMZ4 bulk glass metal. The U-FAST method enabled the production of fully amorphous samples with 100% relative density when sintering at 420 °C/480 s and 440 °C/60 s (693 K/480 s and 713 K/60 s).

The hardness values of fully amorphous samples were over 519 HV1 and are higher than values in the manufacturer materials datasheet for AMZ4 material.

The advantage of the U-FAST technology in this work is attributed to the high heating and cooling rates inherent in ultra-short pulses, which allow to maintain a metastable structure and achieve better temperature control during the process.

Sintering conditions for complex geometry compacts may differ from discs or cylinders of similar diameters and heights. The results of this work suggest that the geometry and material of the dies and punch determine the thermal inertia and pressure distribution inside the compacts. However, this test has shown that it is possible to sinter complex shapes made of metallic glasses using U-FAST.

## Figures and Tables

**Table 1:** Crystallization enthalpies and peak temperatures.

Sample	Crystallization Enthalpy (J/g)	Crystallization Temperature (Peak) (°C)
Powder	57.6	500
420 °C/480 s	56.4	496
440 °C/480 s	13.7	x
460 °C/480 s	4.7	x
440 °C/60 s	56.4	496
460 °C/60 s	41.7	480
Gear wheel	0	x

**Table 2:** Physical and mechanical properties of samples.

Sample	Density (g/cm^3^)	Relative Density (%)	Amorphous Content (%)	Hardness (HV1)	Compressive Strength (MPa)
380 °C/480 s	4.563	68.2%	~100%	x	x
400 °C/480 s	5.747	85.9%	~100%	x	x
420 °C/480 s	6.658	99.5%	~100%	519 ± 13	1551 ± 39
440 °C/480 s	6.766	101.1%*	24	610 ± 3	1097 ± 54
460 °C/480 s	6.779	101.35%*	8	633 ± 8	1052 ± 85
440 °C/60 s	6.691	~100%	~100%	519 ± 5	1625 ± 31
460 °C/60 s	6.712	100.3%*	72	554 ± 13	1409 ± 46
Heraeus Cast sample	6.68	100%	100%	480	1700

* As crystallized material has higher absolute density than the glassy sample the values in the Table tend to be above 100%.

## Data Availability

Data available on request due to restrictions eg privacy or ethical. The data presented in this study are available on request from the corresponding author. The data are not publicly available due to company secret.
